# Seeking Best Practices: A Conceptual Framework for Planning and Improving Evidence-Based Practices

**DOI:** 10.5888/pcd10.130186

**Published:** 2013-12-12

**Authors:** Lorine M. Spencer, Michael W. Schooley, Lynda A. Anderson, Chris S. Kochtitzky, Amy S. DeGroff, Heather M. Devlin, Shawna L. Mercer

**Affiliations:** Author Affiliations: Michael W. Schooley, Chris S. Kochtitzky, Amy S. DeGroff, Heather M. Devlin, Shawna L. Mercer, Centers for Disease Control and Prevention, Atlanta, Georgia; Lynda A. Anderson, Emory University, Atlanta, Georgia.

## Abstract

How can we encourage ongoing development, refinement, and evaluation of practices to identify and build an evidence base for best practices? On the basis of a review of the literature and expert input, we worked iteratively to create a framework with 2 interrelated components. The first — public health impact — consists of 5 elements: effectiveness, reach, feasibility, sustainability, and transferability. The second — quality of evidence — consists of 4 levels, ranging from weak to rigorous. At the intersection of public health impact and quality of evidence, a continuum of evidence-based practice emerges, representing the ongoing development of knowledge across 4 stages: emerging, promising, leading, and best. This conceptual framework brings together important aspects of impact and quality to provide a common lexicon and criteria for assessing and strengthening public health practice. We hope this work will invite and advance dialogue among public health practitioners and decision makers to build and strengthen a diverse evidence base for public health programs and strategies.

## Introduction

In an ideal world, decision makers and practitioners would have access to evidence-based programs and strategies to improve health and reduce health problems with the highest preventable burden. Decision makers would also have tools to guide their selecting and adapting appropriate approaches for specific contexts. In reality, much work needs to be done to achieve this goal, and many pressing public health problems exist where evidence is not yet fully established. Moreover, public health agencies at all levels face substantial fiscal constraints and challenges to improving health across varying contexts. Therefore, the need grows for evidence-based practices that afford maximum efficiency and effectiveness. Many agencies and organizations in the United States have acknowledged such challenges, including the White House (1), the Government Accountability Office (2), the US Department of Health and Human Services (3), the Congressional Budget Office (4), Healthy People 2020 (5), the Community Preventive Services Task Force (6), the National Academy of Sciences (7), and the Trust for America’s Health (8). International attention on evidence-based practice has mirrored that in the United States, including acknowledgment by the United Nations (9) and the World Health Organization (10).

Systematic reviews involve a critical examination of studies addressing a particular issue, using an organized method for locating and evaluating evidence. Many systematic reviews typically favor what Chen (11) refers to as the “Campbellian validity typology” — the use of research designs and methods that maximize internal validity to assess program efficacy. Identifying evidence-based practices by using this paradigm alone will often ignore factors critical to the successful integration of social and public health programs in real world settings. One exception is The Guide to Community Preventive Services (The Community Guide) — a repository of 1) recommendations and findings made by the independent, nonfederal Community Preventive Services Task Force about the effectiveness of community-based programs, services, and policies; and 2) the systematic reviews on which these recommendations are based (www.thecommunityguide.org) (6). The Community Guide reviews include research-tested and practice-based studies that use a range of study designs and assess both internal and external validity (6,12).

There is a need to assess the evidence for, categorize, and encourage additional study of strategies without enough evidence to undergo review by The Community Guide but that hold potential for public health impact. Practitioners and decision makers need tools to guide them toward selecting and evaluating the best available practices when published best practices are unavailable. Although systematic reviews identify what many public health professionals consider best practices, the public health field lacks a consensus definition and commonly accepted criteria for the designation *best practice*.

Consequently, the Office for State, Tribal, Local and Territorial Support at the Centers for Disease Control and Prevention (CDC) convened the CDC Best Practices Workgroup to develop a working definition of *best practices*, along with criteria and processes for classifying diverse practices in relationship to best practice. Through these efforts, the workgroup also aimed to encourage further development of practices that show promise for improving public health outcomes. We describe the development, structure, and use of the workgroup’s conceptual framework for creating a set of best practices. Our purposes in presenting the framework are to promote dialogue among scientists and practitioners about a consistent taxonomy for classifying the evidence for public health practices and to help researchers, practitioners, and evaluators show how their work contributes to building the evidence base for particular practices.

## Background and Approach

The CDC Best Practices Workgroup (see list of members in “Acknowledgments”) consisted of 25 CDC staff members with varying backgrounds (eg, epidemiology, behavioral science, program design and management, evaluation, policy development) and topic expertise (eg, chronic disease, infectious disease, injury prevention, environmental health). The workgroup reviewed the literature to find models and frameworks for classifying evidence, including best practices. The following question guided the review: What is known about the scope and definitions of best practices? The review included a keyword search of peer-reviewed and gray literature; workgroup members also identified relevant literature. The scope of the review was intentionally broad and included concepts related to evidence-based programs and policies as well as practice-based evidence.

The workgroup defined practice as “field-based or research-tested actions intended to effect a positive change.” Practices could include interventions, programs, strategies, policies, procedures, processes, or activities encompassing the 10 essential public health services and related activities (www.cdc.gov/nphpsp/essentialservices.html). This broad definition of practice includes activities designed to improve specific outcomes (eg, morbidity, mortality, disability, quality of life) or to focus on increasing the effectiveness and efficiency of program operations and services.

The review identified articles describing CDC initiatives that develop and disseminate recommendations for public health practice, such as Diffusion of Effective Behavioral Interventions (DEBI) (http://www.effectiveinterventions.org/en/HighImpactPrevention/Interventions.aspx); documents from The Community Guide (6,12); and non-CDC articles about identifying and using evidence to improve practice (1–5,7–10,13). Terms and descriptions found during the literature search appear in Table 1 (14–19). The workgroup abstracted key concepts, including gaps in defining and evaluating best practices and classifying other practices with varying amounts of evidence. A consensus definition of “best practice” was not found, but common elements were. In particular, we found that “best practice” and related terms do not refer to a static assessment or activity; rather, they refer to where, on a continuum, a particular practice falls at a given time. The review identified multiple ways to characterize this continuum or hierarchy, along with considerable variability in the number of stages or levels and in the rigor of methods used for identifying best practices.

**Table 1:** Terms and Definitions Found During Review of Articles on Identifying and Improving Best Practices

Term	Definition
Evidence-based public health	The development, implementation, and evaluation of effective programs and policies in public health through application of principles of scientific reasoning (14).
Evidence-based medicine	The conscientious, explicit, and judicious use of current best evidence in making decisions about the care of the individual patient; integrating individual clinical expertise with the best available external clinical evidence from systematic research (15).
Evidence-based health care	The conscientious use of current best evidence in making decisions about the care of individual patients or the delivery of health services. Current best evidence is up-to-date information from relevant, valid research about the effects of different forms of health care, the potential for harm from exposure to particular agents, the accuracy of diagnostic tests, and the predictive power of prognostic factors (16).
State of the art (SOTA)	SOTA refers to practices that reflect new trends and current thinking in the field. These practices may be successful in localized settings, but much of the evidence is preliminary or anecdotal. A large degree of risk is associated with implementing SOTA practices because they may not have been replicated extensively (17).
Better practice	Better practices are SOTA practices that have been improved on the basis of lessons learned. The projects and interventions show promise for transfer to new settings. Less risk is associated with implementing better practices than with SOTA or innovative practices because of the clearer evidence of success and more lessons learned through experience. Evidence exists in both qualitative and quantitative form but is drawn from applying the practice in limited settings (17).
Research-validated best practice	A program, activity, or strategy that has the highest degree of proven effectiveness supported by objective and comprehensive research and evaluation (18).
Field-tested best practice	A program, activity, or strategy that works effectively and produces successful outcomes and is supported to some degree by subjective and objective data sources (18).
Best practice^a^	A best practice results from a rigorous process of peer review and evaluation that indicates effectiveness in improving public health outcomes for a target population. A best practice 1) has been reviewed and substantiated by experts in the public health field according to predetermined standards of empirical research, 2) is replicable and produces desirable results in various settings, and 3) clearly links positive effects to the program or practice being evaluated and not to other external factors (19).
Promising practice	A program, activity, or strategy that has worked within one organization and shows promise during its early stages for becoming a best practice with long-term, sustainable impact. A promising practice must have some objective basis for claiming effectiveness and must have the potential for replication among other organizations (19).
Emerging practice	Emerging practice 1) incorporates the philosophy, values, characteristics, and indicators of other positive or effective public health interventions; 2) is based on guidelines, protocols, standards, or preferred practice patterns that lead to effective public health outcomes; 3) incorporates a process of continual quality improvement; and 4) has an evaluation plan in place to measure program outcomes, but it does not yet have evaluation data available to demonstrate the effectiveness or positive outcomes (19).
Innovations	Cutting-edge approaches that reflect new, possibly untested thinking. They are sometimes variations on an old theme. Innovations come in the form of pilot programs or experimental projects. Little, if any, objective evidence exists that the practice will have the desired impact (17).

a This definition of best practice informed our proposed definition of best practices: a practice supported by a rigorous process of peer review and evaluation indicating effectiveness in improving health outcomes, generally demonstrated through systematic reviews.

The workgroup created a conceptual framework for planning and improving evidence-based practices by adapting and extending several streams of existing work related to developing a continuum of evidence (6,12,13,20–22). To broaden the framework’s usability, the workgroup developed criteria, definitions, and examples for key terms and formulated a series of questions to apply in assessing and classifying practices. The work was iterative and included frequent comparisons of the framework, definitions, criteria, and assessment questions with how these aspects of public health were discussed in the literature and with the extensive experience of workgroup members. We also continued to refine the products after the workgroup completed its tenure.

## Conceptual Framework

As a result of the review, the workgroup defined the term “best practice” as “a practice supported by a rigorous process of peer review and evaluation indicating effectiveness in improving health outcomes, generally demonstrated through systematic reviews.” The workgroup produced a conceptual framework (Figure) consisting of 2 interrelated components: public health impact and quality of evidence.

**Figure:**
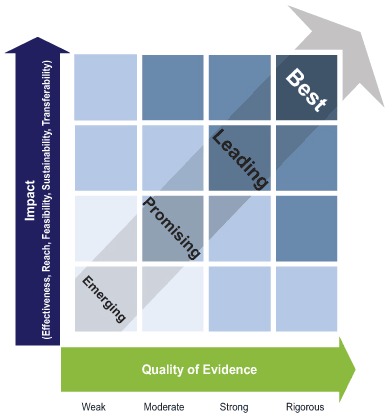
A conceptual framework for planning and improving evidence-based practices.

The public health impact component of the framework consists of the following 5 elements: effectiveness, reach, feasibility, sustainability, and transferability (Box). These elements (represented on the Figure’s vertical axis) are derived in part from the reach, effectiveness, adoption, implementation, maintenance (RE-AIM) model for translational research (20); the integrative validity model (11); and the systematic screening and assessment method (21).

**Box. Definitions for Elements of Public Health Impact and Examples of Questions to Consider Related to the Elements**

**Effectiveness: Extent to which the practice achieves the desired outcomes**

1. What are the practice’s desired outcomes?

2. How consistent is the evidence?

3. What is the magnitude of the effect, including efficiency or effectiveness or both, as appropriate?

4. What is the significance to public health, systems, or organizational outcomes?

5. What are the benefits or risks for adverse outcomes?

6. In considering benefits or risks for adverse outcomes, does the practice promote health equity?

7. To what extent does the practice achieve the desired outcomes?

**Reach: Extent that the practice affects the intended and critical target population(s)**

1. What is the practice’s intended and critical target population (individuals, customers, staff, agency, and other target populations)?

2. What beneficiaries are affected?

3. What is the proportion of the eligible population affected by the practice?

4. How much of the population could ultimately be affected (potential reach)?

5. How representative are the groups that are currently affected compared with groups ultimately affected by the problem?

6. In considering representativeness, does the practice promote health equity?

7. To what extent does the practice affect the intended and critical target population(s)?

**Feasibility: Extent to which the practice can be implemented**

1. What are the barriers to implementing this practice?

2. What are the facilitators to implementing this practice?

3. What resources are necessary to fully implement the practice?

4. Does the practice streamline or add complexity to existing procedures or processes?

5. What is the cost-effectiveness and what are the available resources to implement the practice?

**Sustainability: Extent to which the practice can be maintained and achieve desired outcomes over time**

1. How is the practice designed to integrate with existing programs or processes or both?

2. How is it designed to integrate with existing networks and partnerships?

3. What level of resources is required to sustain the practice over time?

4. What long-term effects or maintenance or improvement of effects over time can be achieved?

5. How has the practice been maintained to achieve its desired outcomes over time?

**Transferability: Extent to which the practice can be applied to or adapted for various contexts**

1. How has the practice been replicated in similar contexts, and did it achieve its intended outcomes?

2. Was adaptation required in different contexts?

3. How has the practice been adapted?

4. What is the impact of varying political, organizational, geographic, social, and economic climates?

5. Has the practice been proven to be effective in different settings?

6. To what extent has the practice been applied to or adapted for a variety of contexts?

Some elements of the public health impact component are more challenging to assess than other elements, such as sustainability and transferability. To address this issue, the workgroup developed questions that users can ask to determine the extent to which the practices they are developing or evaluating address each element (Box). The questions are not comprehensive, and some may not be relevant for all practices; however, having answers to these questions can help facilitate consistent interpretation of impact.

The quality-of-evidence component refers to where a practice lies on an evidence-based practice continuum (Figure). These elements represent 4 levels of evidentiary quality — weak, moderate, strong, and rigorous — that are represented on the horizontal axis. For example, field-based summaries or evaluations of progress with plausible but unproven impact are labeled as weak, whereas assessments of multiple studies or evaluations conducted by using systematic review methods are classified as rigorous (a complete set of definitions and examples is in Table 2) (22).

**Table 2:** Definitions and Examples Related to Levels of the Evidence Quality Supporting Public Health Practices

Level of Evidence	Definitions and Examples
Weak	Field-based summaries or evaluations in progress that have plausible impact (eg, abstracts, book chapters without peer review, demonstration projects lacking appropriate evaluation)
Moderate	Intervention evaluations without peer review of practice or publication that have evidence of impact (eg, case studies with appropriate evaluation, evaluation reports, peer-reviewed abstracts and presentations)
Strong	Case-control or cohort analytic studies; peer-reviewed journal publications; published reports from consensus panels such as the Advisory Committee on Immunization Practices (eg, nonsystematic review of published intervention evaluations with peer review of practices that have evidence of impact)
Rigorous	Intervention evaluations or studies with systematic review that have evidence of impact (eg, meta-analyses, *Guide to Community Preventive Services*)

At the intersection of impact and quality of evidence, a continuum of evidence-based practice emerges, depicted by the arrow at the center of the Figure. This continuum represents the ongoing application of knowledge about what is working to strengthen impact in a given context. Building on the work by Brennan and colleagues (23), a lexicon was created for the continuum consisting of 4 stages — emerging, promising, leading, and best. In this conceptual framework, emerging practices include practices assessed through field-based summaries or evaluations in progress that show some evidence of effectiveness and at least plausible evidence of reach, feasibility, sustainability, and transferability. Emerging practices are generally newer, with a plausible theoretical basis and preliminary evidence of impact. These practices require more implementation and further evaluation to determine whether their potential impact can be replicated over time and in other settings and populations. Promising practices include practices assessed through unpublished intervention evaluations that have not been peer reviewed and that demonstrate some evidence of effectiveness, reach, feasibility, sustainability, and transferability. Promising practices have been evaluated more thoroughly than emerging practices and may include practices with higher quality of evidence and lower impact or with lower quality of evidence and higher impact, where decisions related to application will likely depend on context. Leading practices include practices assessed through peer-reviewed studies or through nonsystematic review of published intervention evaluations that show growing evidence of effectiveness and some combination of evidence of reach, feasibility, sustainability, and transferability. Best practices adhere to the most rigorous assessments in the continuum, including systematic reviews of research and evaluation studies, which demonstrate evidence of effectiveness and growing evidence of reach, feasibility, sustainability, and transferability (eg, The Community Guide [12] and the HIV/AIDS Prevention Research Synthesis Project).

These classifications are hypothesized to be dynamic and can change over time for a given practice. For example, a practice may be assessed as promising at one point and be promoted to leading or best as stronger evidence is developed. Conversely, practices once considered “best” may become outdated as the field and environmental conditions evolve. The dynamic quality of these classifications highlights the importance of regularly re-evaluating practices at all points on the continuum, including updating systematic reviews of best practices. Such updates, incorporating all emerging evidence, are a critical component of the work of the Community Preventive Services Task Force and other review groups (6). By encouraging evaluation at each stage and supporting continued evaluation to move worthy practices up the evidence continuum, the framework aids quality and performance improvement (24).

## Challenges and Next Steps

The framework as it now appears has several limitations: 1) it is conceptual and has not been fully evaluated, and 2) the two axes will be challenging to measure and balance against each other. Classifying a particular practice reliably using the framework depends on the background, perspective, and skill of the individuals assessing the practice.

Because substantial variation exists in practices needed to address various public health challenges, the questions and guidance used to make the designation of emerging, promising, leading, or best require ongoing refinement. Additional tools are needed to guide users in consistently examining the evidence related to a given practice in diverse circumstances. Helping users to develop a clear definition that addresses scope and boundaries for each practice being assessed is important because the development and implementation of public health practices often vary from one setting or population to another. The framework would benefit from additional input by diverse users and stakeholders to inform an iterative process of improvement and refinement over time.

To address these limitations, tools were developed to apply the framework, to determine quality and impact, and to begin to address the complexity of combining 5 elements. The framework and tools are undergoing pilot testing in numerous CDC programs. Pilot testing will assess the validity, intrarater and interrater reliability, and utility of the framework. Results of this pilot testing and further application of the framework will be published.

## Discussion

CDC is congressionally mandated to support the Community Preventive Services Task Force in developing the systematic reviews and related evidence-based recommendations found in The Community Guide (6). CDC also supports development of other systematic review activities (eg, the DEBI project) to identify best practices through rigorous assessment, advance public health science, and promote translation of interventions supported by the highest levels of evidence. The conceptual framework presented here builds on and complements these efforts by offering a practical approach designed for use by public health practitioners, evaluators, and researchers. The framework offers no justification for implementing less than a best practice in cases where a best practice is known and appropriate to address a public health problem. However, in cases where no practices have achieved best practice status, the framework supports the use of the best available practice for a given health problem and context while 1) making sure users recognize where that practice sits on the evidence continuum and 2) encouraging evaluation of impact, including publication of findings to contribute to the overall evidence base for that practice. A key goal for this framework is to improve public health programs by building practice-based evidence (20).

The value of the framework lies in offering a common lexicon and processes to strengthen the evidence for public health practice. The Federal Interagency Workgroup for Healthy People 2020 has adapted the evidence continuum components of the framework as developed by the CDC workgroup. Other stakeholders may use the framework, definitions, and criteria to communicate more clearly about the continuum of evidence for a wide spectrum of programs, including those that do not currently qualify as best practices but may benefit from additional study. Initial feedback from scientists, evaluators, and practitioners, both internal and external to CDC, supports both the face validity of and the need for this framework.

Applying the elements identified in the conceptual framework might assist with program planning, evaluation, and dissemination. The framework could potentially increase transparency and accountability for public health programming by clarifying the components and definitions of evidence. It could help practitioners and evaluators identify gaps in the evidence for a given practice and determine how their work contributes to building the evidence base. Practitioners might attend to the framework’s effectiveness criteria as part of logic model development to help identify potential gaps between activities and intended outputs and outcomes. Several of these criteria already have been adopted in protocols to identify practice-based strategies for more rigorous evaluation (21). The framework’s inclusion of feasibility and transferability could help facilitate adoption by increasing the likelihood that a practice will translate to other settings.

The conceptual framework is offered to promote dialogue among researchers, evaluators, practitioners, funders, and other decision makers. With this framework, we seek to foster a shared understanding for assessing standards of evidence and motivate organizations to implement and improve strategies that move practices along the continuum from emerging to best.

## References

[1] Executive order 13576 — delivering an efficient, effective, and accountable government. Washington (DC): The White House; 2011. http://www.whitehouse.gov/the-press-office/2011/06/13/executive-order-13576-delivering-efficient-effective-and-accountable-gov. Accessed May 13, 2013.

[2] Bascetta CA . Childhood obesity: most experts identified physical activity and the use of best practices as key to successful programs. Washington (DC): US Government Accountability Office; 2005. http://www.gao.gov/assets/100/93790.pdf. Accessed May 13, 2013.

[3] HHS strategic plan. Washington (DC): US Department of Health and Human Services, 2011. http://www.hhs.gov/secretary/about/introduction.html. Accessed May 13, 2013.

[4] Orszag PR . Increasing the value of federal spending on health care. Washington (DC): Congressional Budget Office; 2008. http://www.cbo.gov/sites/default/files/cbofiles/ftpdocs/95xx/doc9563/07-16-healthreform.pdf. Accessed May 13, 2013.

[5] Secretary’s Advisory Committee on National Health Promotion and Disease Prevention Objectives for 2020. Evidence-based clinical and public health: generating and applying the evidence. Washington (DC): US Department of Health and Human Services; 2010. http://healthypeople.gov/2020/about/advisory/EvidenceBasedClinicalPH2010.pdf. Accessed May 13, 2013.

[6] Community Preventive Services Task Force. Annual report to Congress and to agencies related to the work of the Task Force 2013. Atlanta, Georgia. http://www.thecommunityguide.org/annualreport/2013-congress-report-full.pdf. Accessed August 8, 2013.

[7] National Research Council. For the public’s health: the role of measurement in action and accountability. Washington (DC): National Academies Press; 2011. http://books.nap.edu/catalog.php?record_id=13005. Accessed May 13, 2013. 24983050

[8] A healthier America: a new vision and agenda. Washington (DC): Trust for America’s Health; 2007. http://healthyamericans.org/assets/files/WorkingPaper092407.pdf. Accessed May 13, 2013.

[9] Habitat Agenda. The Habitat agenda goals and principles, commitments and the global plan of action. Nairobi (KE): UN-Habitat; 2003. http://www.unhabitat.org/downloads/docs/1176_6455_The_Habitat_Agenda.pdf. Accessed May 13, 2013.

[10] World Health Organization’s Regional Office for Africa. Guide for documenting and sharing “best practices” in health programmes. Brazzaville (CD): World Health Organization; 2008. http://afrolib.afro.who.int/documents/2009/en/GuideBestPractice.pdf. Accessed May 13, 2013.

[11] Chen HT . The bottom-up approach to integrative validity: a new perspective for program evaluation. Eval Program Plann 2010;33(3):205–14. 10.1016/j.evalprogplan.2009.10.002 19931908

[12] Briss PA , Zaza S , Pappaioanou M , Fielding J , Wright-De Aqüero L , Truman BI , Developing an evidence-based Guide to Community Preventive Services — methods. Am J Prev Med 2000;18(1 Suppl):35–43. 10.1016/S0749-3797(99)00119-1 10806978

[13] Tabak RG , Khoong EC , Chambers DA , Brownson RC . Bridging research and practice: models for dissemination and implementation research. Am J Prev Med 2012;43(3):337–50. 10.1016/j.amepre.2012.05.024 22898128 PMC3592983

[14] Brownson RC , Baker EA , Leet TL , Gillespie KN , True WR . Evidence-based public health. 2nd edition. New York (NY): Oxford University Press; 2010.

[15] Sackett DL , Straus SE , Richardson WS , Rosenberg W , Haynes RB . Evidence-based medicine: how to practice and teach EBM. 2nd edition. Edinburgh (UK): Churchill Livingstone; 2000:1.

[16] The Cochrane Collaboration. Evidence-based health care and systematic reviews. London (GB): The Cochrane Collaboration. http://www.cochrane.org/about-us/evidence-based-health-care. Updated April 24, 2013. Accessed May 13, 2013.

[17] Oxlund B . Manual on best practices: HIV/AIDS programming with children and young people. Copenhagen (DK); 2005. http://www.safaids.net/files/Manual%20on%20Best%20Practices%20with%20Children%20and%20Young%20People_AIDSnet.pdf. Accessed May 13, 2013.

[18] Head Start. What are best practices. Washington (DC): US Department of Health and Human Services, Administration for Children and Families, Office of Community Services; 2008. http://eclkc.ohs.acf.hhs.gov/hslc/tta-system/operations/fiscal/after-grant/cp/tAreBestPracti.htm. Accessed May 13, 2013.

[19] Association of Maternal and Child Health Programs. Best practices categories and criteria. Washington (DC). http://www.amchp.org/programsandtopics/BestPractices/Pages/BestPracticeTerms.aspx. Accessed May 13, 2013.

[20] Green LW , Glasgow RE . Evaluating the relevance, generalization, and applicability of research: issues in external validation and translation methodology. Eval Health Prof 2006;29(1):126–53. 10.1177/0163278705284445 16510882

[21] Leviton LC , Khan LK , Rog D , Dawkins N , Cotton D . Evaluability assessment to improve public health policies, programs, and practices. Annu Rev Public Health 2010;31:213–33. 10.1146/annurev.publhealth.012809.103625 20235852

[22] Swinburn B , Gill T , Kumanyika S . Obesity prevention: a proposed framework for translating evidence into action. Obes Rev 2005;6(1):23–33. 10.1111/j.1467-789X.2005.00184.x 15655036

[23] Brennan L , Castro S , Brownson RC , Claus J , Orleans CT . Accelerating evidence reviews and broadening evidence standards to identify effective, promising, and emerging policy and environmental strategies for prevention of childhood obesity. Annu Rev Public Health 2011;32:199–223. 10.1146/annurev-publhealth-031210-101206 21219169

[24] Lenaway D , Corso LC , Buchanan S , Thomas C , Astles R . Quality improvement and performance: CDC’s strategies to strengthen public health. J Public Health Manag Pract 2010;16(1):11–3. 10.1097/PHH.0b013e3181c115ee 20009638

